# Substitutions of the S4DIV R2 residue (R1451) in Na_V_1.4 lead to complex forms of paramyotonia congenita and periodic paralyses

**DOI:** 10.1038/s41598-018-20468-0

**Published:** 2018-02-01

**Authors:** Hugo Poulin, Pascal Gosselin-Badaroudine, Savine Vicart, Karima Habbout, Damien Sternberg, Serena Giuliano, Bertrand Fontaine, Saïd Bendahhou, Sophie Nicole, Mohamed Chahine

**Affiliations:** 1 Centre de recherche CERVO, Institut Universitaire en Santé Mentale de Québec, Quebec City, QC G1J 2G3 Canada; 2 Department of Medicine, Université Laval, Quebec City, QC G1K 7P4 Canada; 3 UMR7370 CNRS, LP2M, Labex ICST, University Nice Sophia-Antipolis, Faculté de Médecine, Nice, France; 4 AP-HP, Centre de référence Canalopathies Musculaires, Groupe Hospitalier Pitié Salpêtrière, Paris, France; 5 INSERM, U1127, Sorbonne universités, UPMC University Paris 6, UMR S1127, CNRS, UMR 7225, Institut du Cerveau et de la Moelle épinière, ICM, Paris, France

## Abstract

Mutations in Na_V_1.4, the skeletal muscle voltage-gated Na^+^ channel, underlie several skeletal muscle channelopathies. We report here the functional characterization of two substitutions targeting the R1451 residue and resulting in 3 distinct clinical phenotypes. The R1451L is a novel pathogenic substitution found in two unrelated individuals. The first individual was diagnosed with non-dystrophic myotonia, whereas the second suffered from an unusual phenotype combining hyperkalemic and hypokalemic episodes of periodic paralysis (PP). The R1451C substitution was found in one individual with a single attack of hypoPP induced by glucocorticoids. To elucidate the biophysical mechanism underlying the phenotypes, we used the patch-clamp technique to study tsA201 cells expressing WT or R1451C/L channels. Our results showed that both substitutions shifted the inactivation to hyperpolarized potentials, slowed the kinetics of inactivation, slowed the recovery from slow inactivation and reduced the current density. Cooling further enhanced these abnormalities. Homology modeling revealed a disruption of hydrogen bonds in the voltage sensor domain caused by R1451C/L. We concluded that the altered biophysical properties of R1451C/L well account for the PMC-hyperPP cluster and that additional factors likely play a critical role in the inter-individual differences of clinical expression resulting from R1451C/L.

## Introduction

Voltage-gated Na^+^ channels (VGSC) are responsible for the initial rise and propagation of action potentials (APs) in excitable cells. The *SCN4A* gene encodes the alpha subunit of Na_V_1.4, the skeletal muscle VGSC. Na_V_1.4 is composed of four homologous domains (DI to DIV). Each domain is composed of a Voltage Sensor Domain (VSD, S1 to S4) and a pore domain (S5-S6). The linker between DIII and DIV forms the inactivation gate. One of the most important features of membrane excitability is the availability of VGSC for activation. Membrane excitability is driven by the inactivation process that leads to the closing of VGSC even when the membrane is depolarized. Over 50 *SCN4A* mutations^1^ have been associated with six skeletal muscle Na^+^ channelopathies forming a clinical spectrum ranging from severe forms of Na^+^ channel myotonia (SCM, hyperexcitability) to fetal hypokinesia (hypoexcitability)^2,3^. Among these extreme phenotypes are found the paramyotonia congenita (PMC), periodic paralysis (PP), and congenital myasthenic syndromes (CMS)^2^. All of these channelopathies have an autosomal dominant mode of inheritance except those with congenital muscle weakness (myasthenia, myopathy and hypokinesia) that result from recessive mutations^3–6^.

The physiological basis of non-dystrophic myotonia (NDM) linked to Na_V_1.4 (SCM and PMC) involves the hyperexcitability of the muscle membrane; this causes a delayed relaxation of muscle fibers due to sustained action potentials (APs) firing following voluntary contractions. Individuals complain of muscle stiffness after voluntary effort. In contrast, periodic paralysis (PP) is a transient state of hypoexcitability of the muscle membrane brought on by sustained sarcolemmal depolarization in which APs cannot be triggered or propagated^7^. The hallmark of PP is recurrent episodes of weakness, which may be regional or generalized, and concomitant to reduced (hypokalemic PP) or normal to increased (normo or hyperkalemic PP) level of blood potassium. The severity varies from mild to flaccid paralysis with an inability to sit, stand, or walk. In general, gain-of-function mutations in *SCN4A* that enhance activation and impair inactivation of the mutant Na_V_1.4 channel are associated with SCM and the clinical continuum NDM/hyperPP, respectively, whereas loss-of-function mutations that drastically enhance inactivation are associated with myasthenic and myopathic-like phenotypes^7^. All dominantly-inherited hypoPP phenotypes are due to amino-acid substitution of arginine residues in the segment 4 (S4) of DI-III VSDs that cause a dominant-negative effect with a gating pore current responsible for a proton or sodium leak and paradoxical depolarization of the skeletal muscle membrane.

If the clinical continuity between NDM and hyperPP has been well described, little is known regarding a possible genetically-based clinical overlap between the NDM-hyperPP and hypoPP clusters^1,2^. We report here the functional impact of two dominant *SCN4A* missense mutations that substitute for the same arginine residue in S4 of DIV VSD and are linked to 3 unusual clinical expressions of Na^+^ channelopathies. The previously reported R1451C was found in one individual with glucocorticoid-induced hypoPP, while R1451L was found in one individual diagnosed with cold-sensitive NDM and a second individual presenting with a mixed phenotype of hyper and hypoPP. To elucidate the mechanisms underlying the clinical phenotypes linked to substitution of R1451 residue, we used the whole-cell patch-clamp technique to study tsA201 cells expressing the WT, R1451C or R1451L channels. Our results showed that both substitutions shifted steady-state inactivation to hyperpolarized potentials, reduced the current density, impaired the recovery from slow inactivation and increase the overall inactivation rate.

## Results

### Clinical evaluation

#### Individual 1 with glucocorticoid-induced hypoPP: carries the p.R1451C substitution

The phenotype of individual 1 was described in details elsewhere^8^. Briefly, this 27-year-old man, without familial history, had a single episode of hypokalemic quadriplegia following a glucocorticoid injection done one day after a high carbohydrate meal. He did not report cold-induced stiffness or weakness at the time of examination. He was found to be heterozygous for the c.4351c > t mutation resulting in the p.Arg1451Cys (thereafter named R1451C) amino acid substitution.

#### Individual 2 with cold-induced myotonia: carries the p.R1451L substitution

A 38-year-old male individual was referred to our referent center for myotonia. The family had no particular health issues, except that family members on the maternal side (the patient’s mother and maternal grandfather) reported mild cold-induced muscle stiffness. The patient had never been excluded from sports as a child. During the course of adolescence, he practiced martial arts, cycling, and other sports in school without difficulty until the age of 16–17 years. In one instance, during a shot-put test during the winter (outdoor cold temperatures), his fingers remained tense on the machine, preventing the launch. There was no associated pain. Thereafter, he noticed that the phenomenon occurred every time his hands were exposed to cold temperatures. It also occurred after cold water dives, making it difficult for him to remove his equipment due to painless twitching of his fingers that lasted on average one hour. He felt no discomfort in his face or legs, had no paralytic episodes, and had no triggers other than cold. The cold-sensitivity of stiffness episodes was suggestive of PMC related to a mutation in *SCN4A*. An electromyography (EMG) investigation showed some myotonic bursts and Fournier pattern type III suggestive of SCM^9^. Given that the discomfort was minimal and that the patient had learned to manage the symptoms, there was no need to begin a symptomatic treatment. Sanger sequencing identified a heterozygous c.4352 g > t mutation in *SCN4A* encoding the p.Arg1451Leu (thereafter named R1451L) amino acid substitution in Na_V_1.4.

#### Individual 3 with a mixed phenotype of hyper- and hypo-PP: carries the p.R1451L substitution

The patient was a 23-year-old male. The symptoms began at age 10 years, with transient episodes of weakness of the lower limbs of varying duration (several minutes to several hours) that were spontaneously regressive and that mainly occurred in the late morning at school, preventing him from leaving his chair. Paralytic episodes became more frequent over the years, leading to regular hospitalizations. His serum potassium levels always remained within normal limits. A more severe paralytic episode than usual affected all four members following an unusual and sustained physical effort at age 17. EMG investigations were suggestive of hyperPP with the presence of myotonic bursts and a delayed fall in AP amplitudes after a sustained effort (Fournier pattern type IV)^10^. Accordingly, potassium load for diagnosis purpose resulted in one attack of PP. Three years later, he woke up quadriplegic after a party with a carbohydrate rich meal the preceding night. His serum potassium levels were below the normal limits (2 mmol/l), suggestive of hypokalemic episode of PP. The symptoms gradually decreased over 48 h following an intravenous potassium recharge. The patient thus exhibited a combination of hyperkalemic and hypokalemic episodes of paralysis. Diamox and Diffu-K were prescribed to prevent further paralytic attacks. The patient’s father was afterward reported to suffer from muscle weakness of the lower limbs after minor efforts, but the hyper or hypokalemic type of the episodes could not be documented. Genetic testing revealed a paternally inherited heterozygous c.4352 g > t mutation in *SCN4A* resulting in R1451L in Na_V_1.4.

### Current densities, activation and fast inactivation

**Figure 1:**
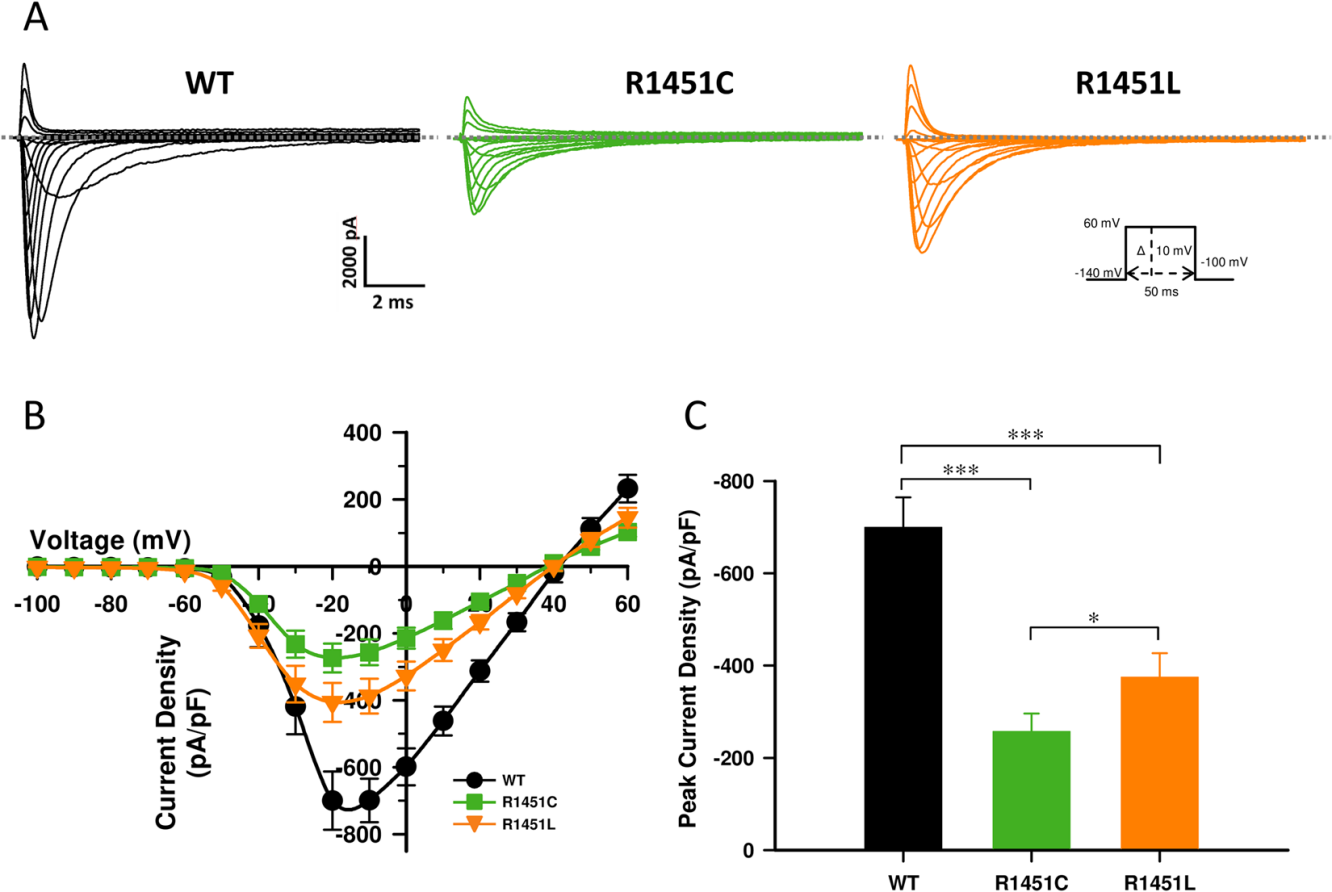
Whole-cell properties of the WT, R1451C, and R1451L channels. (**A**) Representative whole-cell traces recorded from tsA201 cells expressing WT, R1451C, or R1451L channel. Currents were elicited from a holding potential of −140 mV and were depolarized to potentials ranging from −140 to +60 mV in 10-mV increments in 50-ms steps (see protocol in the inset under the R1451L traces). The dashed line represents zero current. (**B**) Normalized current/voltage relationships of the WT (*n* = 10), R1451C (*n* = 18), and R1451L (*n* = 18) channels. Current densities were measured by normalizing current amplitudes to membrane capacitance and were plotted against voltage. The experimental protocol was the same as in (**A**). (**C**) Histogram showing the average peak current densities recorded at −10 mV of the WT, R1451C, and R1451L channels. The R1451C and R1451L current densities decreased by 63% (***P < 0.001) and 46% (***P < 0.001), respectively, compared to the WT channel. R1451C current density decreased by 31% (*P < 0.05) compared to the R1451L channel.

Although previously reported, the R1451C mutation has never been biophysically characterized. We compared this substitution to p.R1451L in order to study if the nature of the R1451 substitution could translate to a specific clinical feature. The biophysical properties of the WT, R1451C, and R1451L VGSC were studied in a heterologous expression system. Na^+^ current recordings (Fig. 1A) and current-density relationships (Fig. 1B) were obtained from tsA201 cells transfected with WT, R1451 C, or R1451L human cDNAs. The current densities of the R1451C (257.0 pA/pF) and R1451L (374.5 pA/pF) channels were reduced by 63% and 46%, respectively, compared to the WT (699.3 pA/pF) channel (Fig. 1C).

**Figure 2:**
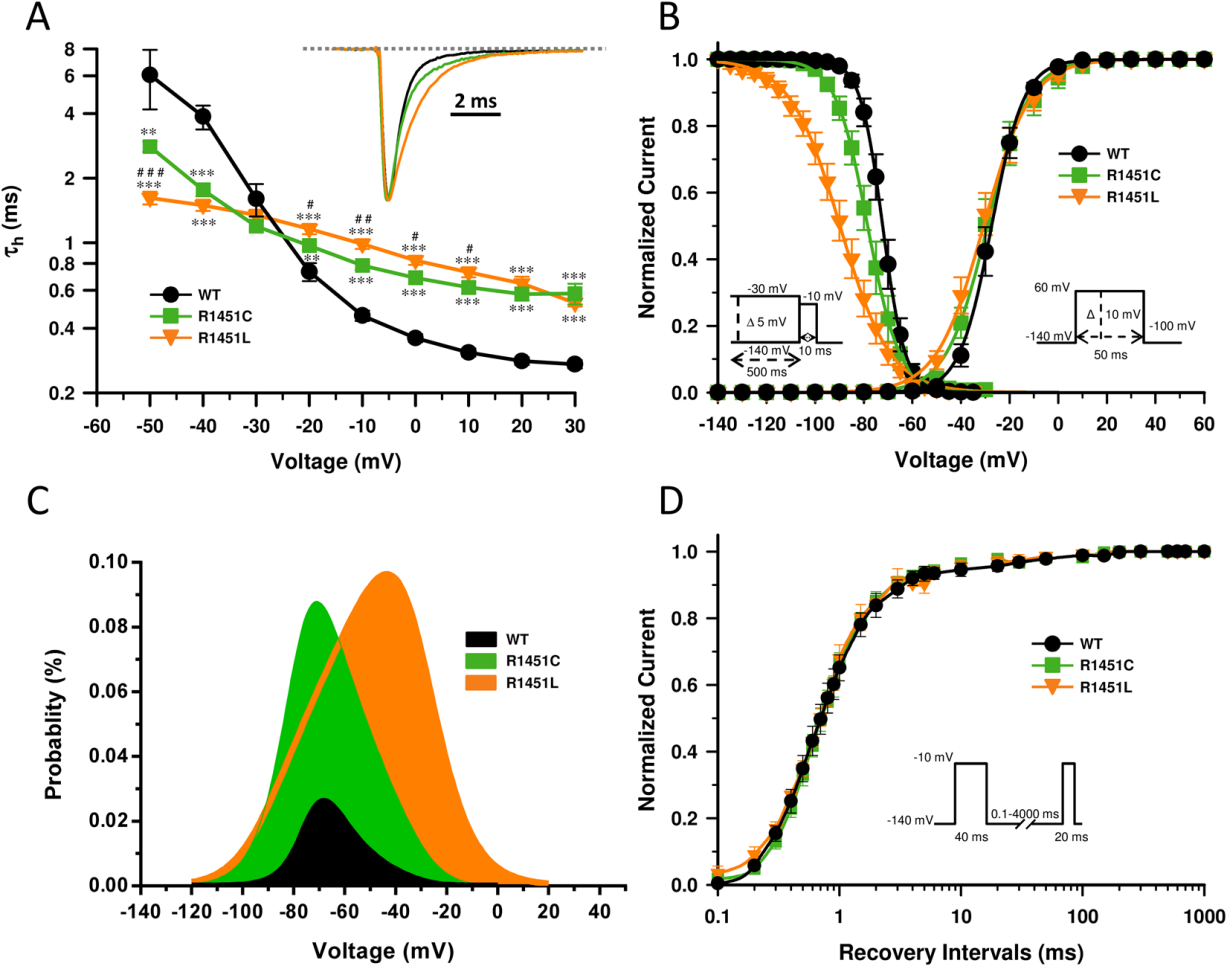
Gating properties of the channels. (**A**) The voltage dependence of the inactivation time constants of the WT (*n* = 9), R1451C (*n* = 8), and R1451L (*n* = 15) channels. The time course of the current decay elicited at depolarized voltages was best fitted to a single exponential function, and the resulting time constants (*τ*_*h*_) were plotted against voltage. The kinetics of fast inactivation were slower for the R1451C and R1451L channels, with R1451L being the slowest. The inset shows representative normalized Na^+^ currents elicited at −10 mV. The statistical significances of the differences between WT and R1451C/L (**P < 0.001, ***P < 0.001) and between R1451C and R1451L (^#^*P* < 0.05, ^##^*P* < 0.001, ^###^*P* < 0.001) are shown. (**B**) Steady-state activation and inactivation of the WT and the mutants. No significant shifts in the V_1/2_ of the R1451C/L channels were observed. Steady-state inactivation was determined using 10 ms test pulses to −10 mV after a 500-ms pre-pulse to potentials ranging from −140 mV to −30 mV (see protocol in the inset under the inactivation curves). The inactivation curves of R1451C and R1451L channels were left-shifted by −6 mV and −20 mV, respectively. The V_1/2_, the k_v_ and the *n* values are given in Table 1. (**C**) Effect of R1451C/L on Na_V_1.4 window current. The probability of being within this window was calculated through the following equation: (1/{1 + exp[(V_1/2act_ − V)/k_act_]} × ((1 − C)/{1 + exp[(V − V_1/2inact_)/k_inact_]} + C). R1451C/L mutations produced a 5 and 7-fold increase in the window current, respectively (**D**) Recovery from inactivation of WT (*n* = 5), R1451C (*n* = 8) and R1451L (*n* = 6) channels. The cells were depolarized to −10 mV for 40 ms from a holding potential of −140 mV to inactivate the Na^+^ channels. Test pulses were then applied to −10 mV for 20 ms to measure current amplitudes, with an interval ranging from 0.1 to 4000 ms. The resulting curves were fitted to a single exponential equation. No significant effect was observed. The values are given in Table 1.

A common feature of most pathogenic amino acid substitutions of Na_V_1.4 associated with the clinical continuum PMC/hyperPP is inactivation defects whereas SCM result from activation defects^1^. The kinetics of fast inactivation from the open state were analyzed by fitting the decay of Na^+^ whole-cell currents elicited at voltages from −50 to +30 mV with a single exponential time course to verify the integrity of inactivation kinetics. Representative Na^+^ currents elicited at −10 mV are shown in Fig. 2A (inset). Compared to WT channels, the time constants (*τ*_*h*_) were significantly larger for R1451C and R1451L channels, R1451L being the most affected (Fig. 2A). For example, at −10 mV, the *τ*_*h*_ of Na^+^ currents of WT, R1451C and R1451L channels were 0.46 ± 0.03, 0.78 ± 0.04 and 0.99 ± 0.05 ms, respectively. The R1451L substitution also promoted a loss of voltage-dependence (for entry into fast inactivation), especially at hyperpolarized voltages. No persistent Na^+^ current were observed (data not shown).

Fast inactivation is a process by which VGSC switch to a non-conducting state in milliseconds after the onset of depolarization. This process contributes to the repolarization of the AP. Figure 2B shows that R1451C and R1451L mutations caused a shift of 6.0 and 19.6 mV, respectively, in the midpoint (V_1/2_) of steady-state inactivation in a hyperpolarizing direction (WT: V_1/2_ = −72.2 ± 1.4 mV, R1451C: V_1/2_ = −78.2 ± 2.1 mV, R1451L: V_1/2_ = −91.8 ± 3.3 mV; Table 1). In addition, the steepness of the inactivation curves was lower for the R1451C and R1451L channels compared to the WT channel, R1451L being the most affected (WT: *k*_v_ = 4.2 ± 0.1 mV, R1451C: *k*_v_ = 5.9 ± 0.2 mV, R1451L: *k*_v_ = 9.4 ± 0.3 mV; Table 1).

**Table 1:** Biophysicals properties of WT, R1451C and R1451L channels at 22 °C.

	WT		R1451C		R1451L	
	Mean ± sem	*n*	Mean ± sem	*n*	Mean ± sem	*n*
**Steady-state activation**		9		8		15
*V*_*1/2*_	−27.5 ± 1.8		−28.8 ± 2.8		−30.7 ± 2.5	
*k*_*v*_	−5.8 ± 0.3		−7.6 ± 0.5**		−7.9 ± 0.3***	
**Steady-state inactivation**		7		8		9
*V*_*1/2*_	−72.2 ± 1.4		−78.2 ± 2.1*		−91.8 ± 3.3***	
*k*_*v*_	4.2 ± 0.1		5.9 ± 0.2***		9.4±0.3***	
**Recovery from fast inactivation**		5		8		6
τ	1.1 ± 0.1		1.1 ± 0.1		1.3 ± 0.1	
**Onset of slow inactivation**		10		7		8
τ_fast_	39 ± 9.5		36 ± 11		38 ± 16	
*A*_fast_	6.4 ± 1.5		10 ± 1.4		7.9 ± 0.8	
τ_slow_	3810 ± 465		4193 ± 376		3085 ± 345	
**Recovery from slow inactivation (4-s cond. pulse)**		7		6		7
τ_fast_	3.3 ± 0.5		4.2 ± 1.8		2 ± 0.2	
*A*_fast_	47 ± 5.4		43 ± 4.9		41 ± 5.5	
τ_slow_	243 ± 43		291 ± 79		420 ± 105	
**Recovery from slow inactivation (30-s cond. pulse)**		8		5		7
τ_fast_	24 ± 12		59 ± 16		162 ± 46**	
*A*_fast_	46 ± 6.5		54 ± 12		41 ± 8.9	
τ_slow_	382 ± 64		726 ± 97**		1978 ± 206***	

*V*_1/2_, midpoint for activation or inactivation (mV); *k*, slope factor for activation or inactivation; τ, time constant (ms); *A*, fraction of the τ components (%); *n*, number of cells. Values are means ± sem, *P < 0.05, **P < 0.001, ***P < 0.001, data were significantly different for mutant channels when compared to WT at 22 °C.

The effects of R1451C and R1451L mutations on steady-state activation were investigated (Fig. 2B). The current voltage relationship (I/V curves) were converted to conductance against voltage (G/V) curves and were fitted using a Boltzmann function (see Methods). The V_1/2_ of the voltage-dependence of activation was unaffected by R1451C and R1451L mutations (WT: V_1/2_ = −27.5 ± 1.8 mV, R1451C: V_1/2_ = −28.8 ± 2.8 mV, R1451L: V_1/2_ = −30.7 ± 2.5 mV; Table 1). In contrast, the *k*_*v*_ values of the mutant channels were slightly higher, resulting in activation curves less steep than that of WT channels (WT: *k*_v_ = −5.8 ± 0.3 mV, R1451C: *k*_v_ = −7.6 ± 0.5 mV, R1451L: *k*_v_ = −7.9 ± 0.3 mV; Table 1). The time courses of recovery from fast inactivation of the WT, R1451C, and R1451L channels can be described by a single exponential function (Fig. 2D). The kinetics of the recovery from inactivation of the WT and mutant channels were similar (Table 1).

The overlap of steady-state activation and inactivation defines a range of voltages (i.e. window) where the probability of VGSC opening is significant. Figure 2C shows the predicted windows currents of WT and R1451C/L mutant channels. The R1451C and R1451L mutations produced a ∼5 and 7-fold increase in the Na_V_1.4 window current, respectively, suggesting that in the presence of these mutations more channels may reopen within the window region especially near the resting membrane potential.

### Slow inactivation

**Figure 3:**
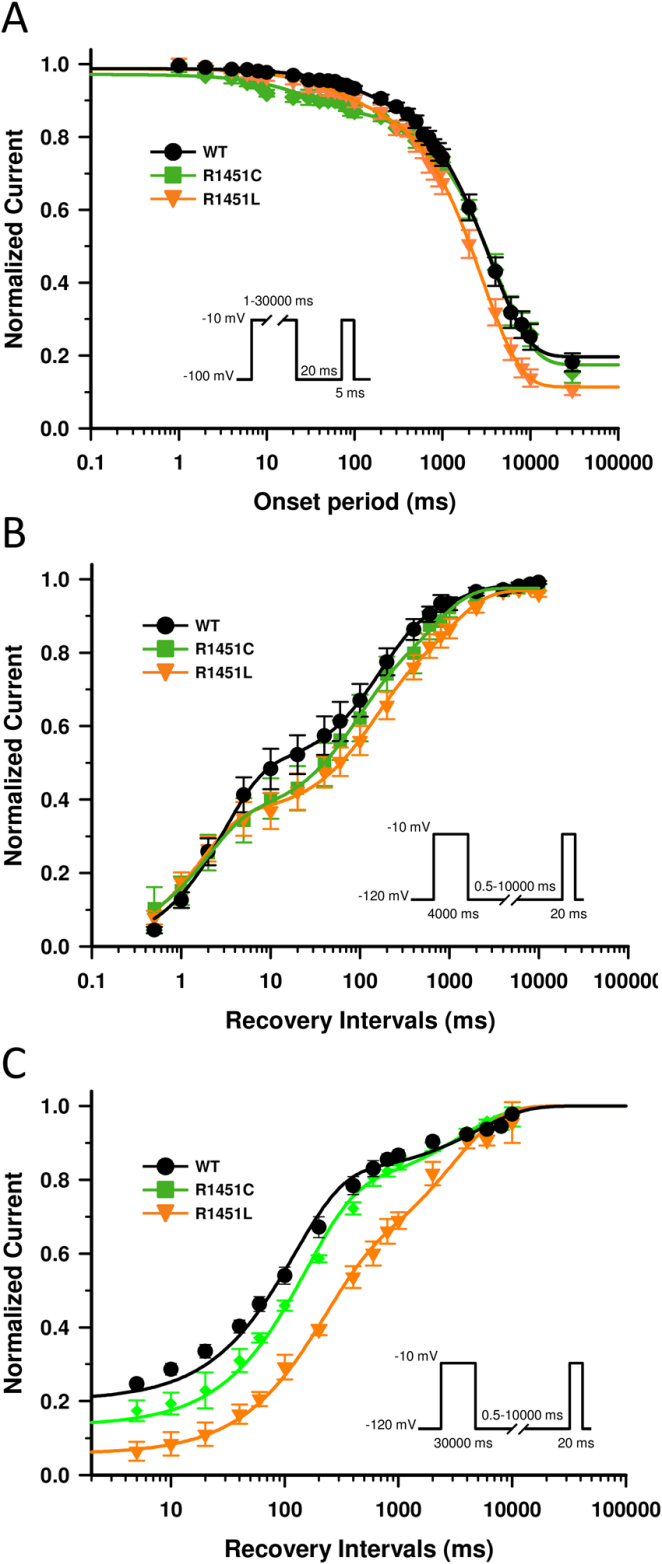
Slow inactivation properties of WT, R1451C and R1451L channels. (**A**) Onsets of slow inactivation of WT (*n* = 10), R1451C (*n* = 7), and R1451L (*n* = 8) channels. The entry into slow inactivation was measured using a double-pulse protocol. The cells were first depolarized to −10 mV for a variable duration (1 ms to 30 s) from a holding potential of −120 mV. Following a 20-ms interpulse at −120 mV to allow complete recovery from fast inactivation, a brief 5-ms test pulse to −10 mV was applied to measure the available Na^+^ current (fraction not slow inactivated). The resulting curves were fitted to a double exponential equation, which yielded two time constants: τ_fast_ and τ_slow_. There was no significant difference in the time constants of WT, R1451C, and R1451L channels. (**B**,**C**) Recovery from slow inactivation of WT (*n* = 7), R1451C (*n* = 6), and R1451L (*n* = 7) channels. The cells were depolarized to −10 mV for 4 s (**B**) or 30 s (**C**) from a holding potential of −120 mV to inactivate all Na^+^ channels. Test pulses were then applied for 20 ms to −10 mV to measure current amplitudes, with an interval ranging from 0.5 ms to 10 s. The resulting curves were fitted to a double exponential equation, which yielded two time constants: τ_fast_ and τ_slow_. After a short (4 s) conditioning pulse, there was no significant difference in the time constants of WT, R1451C and R1451L channels. After a longer (30 s) conditioning pulse, τ_slow_ of R1451C (726 ± 97 ms) and R1451L (1978 ± 206 ms) is larger than τ_slow_ of WT (382 ± 64 ms).

Slow inactivation is a universal feature of VGSC whereby, after a prolonged depolarization, they close off via a process that is completely different from fast inactivation. Slow inactivation defects associated with myotonia/paralytic phenotypes have been found in several Na_V_1.4 mutant channels^11,12^. We evaluated the onset and recovery from slow inactivation of WT, R1451C and R1451L channels with a sequential-pulse protocol (Fig. 3), and fitted the data using an exponential function with a smaller (*τ*_*fast*_) and a larger (*τ*_*slow*_) components. The rates of entry to slow inactivation of mutant channels were similar to those of WT channels (Fig. 3A, Table 1). We also measured the recovery from intermediate or slow inactivation following a 4-s or 30-s conditioning pulse at −10 mV, respectively (Fig. 3B and C). The data were plotted and fitted using an exponential function with smaller (*τ*_*fast*_) and larger (*τ*_*slow*_) time constants (Fig. 3B and C). After a 30-s conditioning pulse, the recovery of R1451C and R1451L channels were about 2 and 5 times longer than WT channels, respectively, as shown by *τ*_*slow*_ (WT: 382 ± 64 ms, R1451C: 726 ± 97 ms, R1451L: 1978 ± 206 ms; Table 1). The *τ*_*fast*_, which accounts for ∼40 to ∼50% of the exponential components, was 7 times longer in R1451L than WT channels, but was not significantly affected in R1451C channels (WT: 24 ± 12 ms, R1451C: 59 ± 16 ms, R1451L:162 ± 46 ms; Table 1). After a 4-s conditioning pulse, the recovery from intermediate inactivated state was unaffected in mutant channels compared to WT channels (Fig. 3B and Table 1).

### Effect of temperature

**Figure 4:**
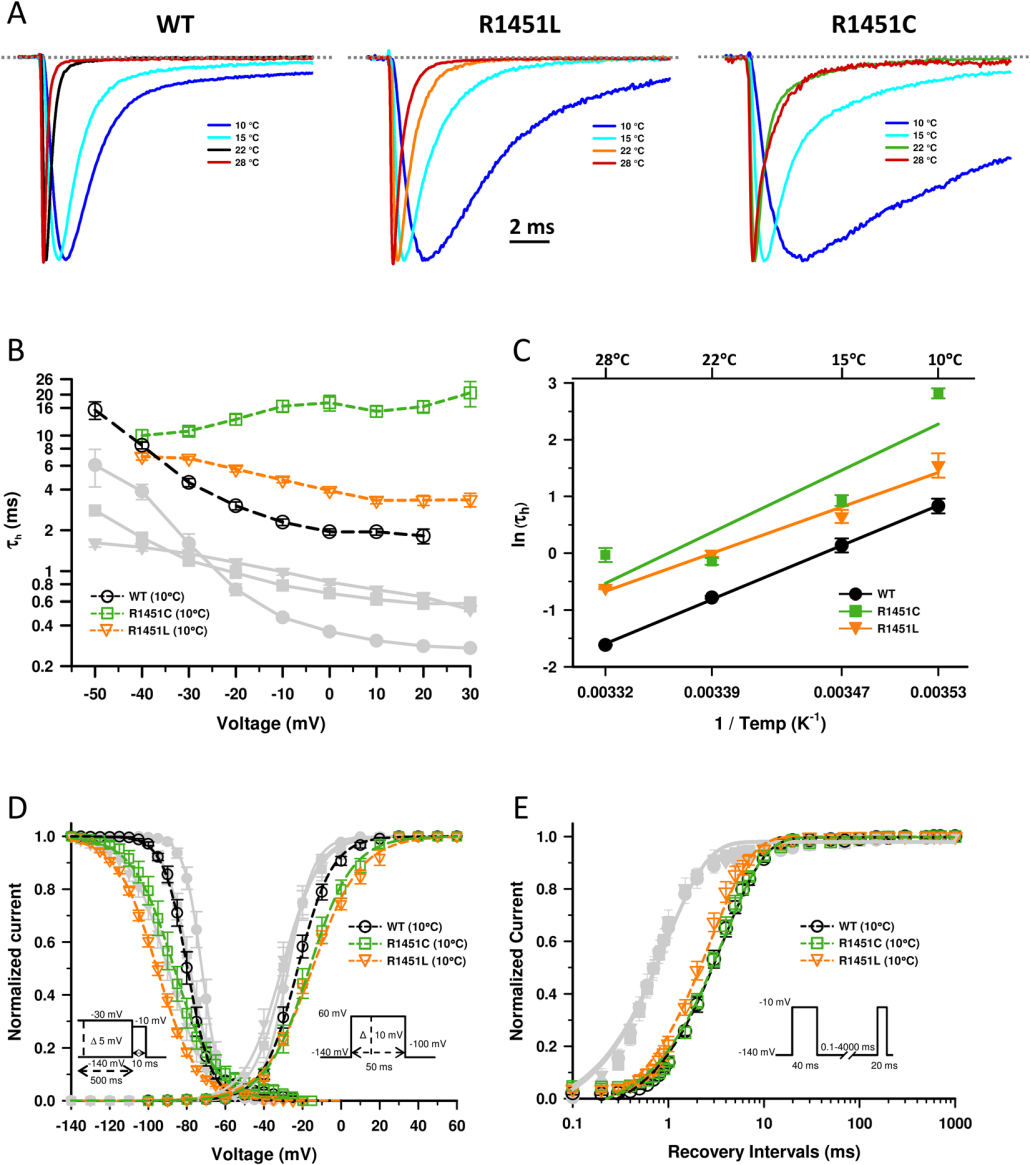
Effect of temperature on WT, R1451C and R1451L channels. (**A**) Representative whole-cell traces recorded at −10 mV of WT, R1451L and R1451C channels at 10 °C (blue), 15 °C (purple), 22 °C (black/orange/green) and 28 °C (red). The dashed line represents zero current. (**B**) Voltage dependence of inactivation time constants of WT (*n* = 9), R1451C (*n* = 8) and R1451L (*n* = 7) channels recorded at 10 °C. The time course of the current decay elicited at depolarized voltages was best fitted to a single exponential function, and the resulting time constants (τ_h_) were plotted against voltage. The kinetics of fast inactivation were slower for all channels at all voltages by cooling to 10 °C. The statistical significances of the differences between the WT and R1451C/L channels (*P < 0.05, **P < 0.001, ***P < 0.001) are shown. (**C**) Arrhenius plots showing the temperature sensitivities of WT and R1451C/L channels. Unlike R1451C, the slope of the R1451L was significantly different from that of the WT as revealed by ANCOVA statistical test (**P < 0.005). The R1451 Each data point is a mean of 6–15 cells. (**D**) Steady-state inactivation (left curves) after a 500-ms prepulse was left-shifted for WT (*n* = 10), R1451C (*n* = 8) and R1451L (*n* = 7) by 7, 8 and 5 mV, respectively, whereas that of steady-state activation (right curves) was right-shifted by 5, 12 and 16 mV for WT (*n* = 11), R1451C (*n* = 8) and R1451L (*n* = 9), respectively. The V_1/2_, k_v_, and *n* values are given in Table 2. The protocols are the same as those in Fig. 2C. (**E**) Recovery from inactivation of WT (*n* = 5), R1451C (*n* = 8) and R1451L (*n* = 5) channels. The resulting curves were fitted to a single exponential equation, yielding one time constant (τ). The τ of R1451L (3.3 ± 0.3 ms) was slightly smaller than the τ of WT channel (4.4 ± 0.3 ms). The values of the time constants are given in Table 2. For clarity, the grey curves in (**B**,**D** and **E)** represent the experiments performed at 22 °C (see Fig. 2).

One of the distinctive clinical features of individuals with PMC is their sensitivity to cold. Cold sensitivity was documented for individual 2 with the R1451L mutation but not for individual 3 carrying the same mutation. Although individual 1 did not report cold sensitivity at the time of last examination, the effect cooling on R1451C was also investigated. Figure 4A show representative current traces of WT, R1451C and R1451L channels recorded at 10 °C, 15 °C, 22 °C and 28 °C. A decrease in temperature produced a general slowing of kinetics at all voltages for WT and R1451C/L channels (Fig. 4B). Sensitivity of this response was quantified in an Arrhenius plot, a plot of the log of the measured parameter against 1/Temp (°K) (Fig. 4C). This plot is a direct measure of temperature sensitivity, since the slope of the plot reflects the change in *τ*_*h*_ parameter in function of temperature. While there was no significant change of R1451C, the slope obtained for R1451L was significantly reduced compared to WT, giving energies of activation equal to 19.9 ± 1.5 (WT), 21.3 ± 2.3 (R1451L) and 22.9 ± 1.4 (R1451C) kcal/mol. Thus, the R1451L mutation produces a small decrease in the temperature sensitivity of fast inactivation. The steady-state inactivation of WT, R1451C and R1451L were shifted to hyperpolarized potentials by 7, 8 and 5 mV when cooling from 22 °C to 10 °C, respectively (Fig. 4D, Table 2). The kinetics of recovery from fast inactivation of WT and R1451C channels were slowed in a similar manner as shown by the increase of *τ*_*fast*_, while R1451L was less affected by cooling from 22 °C to 10 °C, (Fig. 4E, Table 2). Steady-state activation was also studied at 10 °C. Figure 4D shows that the steady-state activation of R1451C and R1451L was more affected by cooling than that of WT channels. While there was no significant shift at 22 °C, the V_1/2_ of R1451C and R1451L was shifted respectively by 6 and 7.8 mV toward depolarized potentials at 10 °C compared to WT (Fig. 4D, Table 2).

**Table 2:** Biophysicals properties of WT, R1451C and R1451L channels at 10 °C.

	WT		R1451C		R1451L	
	Mean ± sem	*n*	Mean ± sem	*n*	Mean ± sem	*n*
**Steady-state activation**		11		8		9
*V*_*1/2*_	−22.5 ± 1.6		−16.5 ± 2.7*		−14.7 ± 1.3**	
*k*_*v*_	−8.4 ± 0.2		−10.6 ± 0.3***		−12.4 ± 0.7***	
**Steady-state inactivation**		10		8		7
*V*_*1/2*_	−79.7 ± 1.4		−86.4 ± 2.8*		−97 ± 1.2***	
*k*_*v*_	5.7 ± 0.3		10 ± 0.9***		10.2 ± 0.2***	
**Recovery from fast inactivation**		5		8		6
τ_slow_	4.4 ± 0.2		4.3 ± 0.3		3.3 ± 0.3*	

*V*_1/2_, midpoint for activation or inactivation (mV); *k*, slope factor for activation or inactivation; τ, time constant (ms); *A*, fraction of the τ components (%); *n*, number of cells. Values are means ± sem, *P < 0.05, **P < 0.001, ***P < 0.001, data were significantly different for mutant channels when compared to WT at 22 °C.

### Molecular modeling

**Figure 5:**
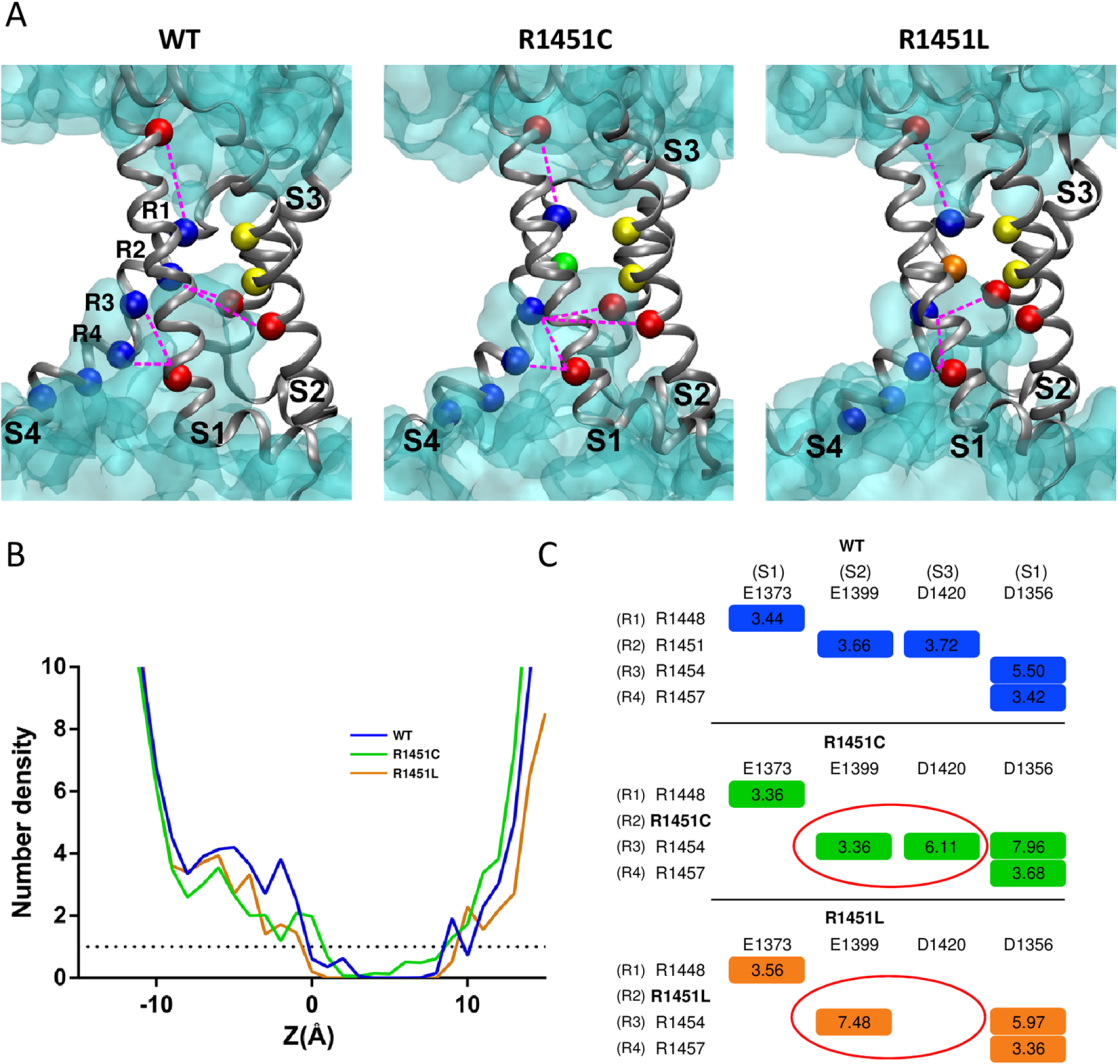
Structural model of the resting state VSD of DIV of the WT, R1451C, and R1451L channels. (**A**) The backbones of the S1, S2, S3, and S4 transmembrane segments are represented as gray ribbons. The positively charged Arginine (R) residues of S4 are represented as blue balls, and the negative counter charges are in red. The conserved residues of S2 that define the hydrophobic seal are in yellow. The R1451C and R1451L substitutions are in green and orange, respectively. The hourglass-like solvent accessible volume is shown as a transparent turquoise surface. The hydrogen bonds between the charged residues are shown as pink dotted lines. For clarity, the dotted lines are between ^α^C of the residues instead of the side chains. (**B**) Chart representing the water profile in the VSD. The number of water molecules is shown as a function of distance, where 0 Å is the center of the membrane. The dotted line indicates the limit of the hydrophobic septum. Less than one water molecule is found below this line. (**C**) Table indicating the hydrogen bonds shown in (**A**) and their distances in Å. Red circles highlight differences between mutant and WT channels.

To investigate the molecular differences between the WT, R1451C and R1451L channels, we built a homology model of the VSD of DIV of Na_V_1.4 in the resting state based on the Na_v_Ab channel X-ray structure (*Acrobacter butzleri* Na^+^ channel)^13^. One characteristic of the VSD is the presence of a constriction site involving a conserved phenylalanine residue in S2 that prevents the intra- and extracellular solutions from communicating at the center of the domain. This hydrophobic septum is essential for the proper functioning of the channel to prevent ions from passing through the VSD during the displacement of S4. As shown by the topology of the solvent in the VSD (Fig. 5A), the hydrophobic septa of R1451C and R1451L channels were similar to that of the WT channel. There were no significant differences in the number of water molecules in relation to the position of the septa in the VSDs (Fig. 5B). The hydrogen bonds between the amino acid residues of the S4 segment and the residues of the other segments were the only differences exposed by the model between WT, R1451C and R1451L channels. As expected, the hydrogen bonds between the R2 residue (R1451) of S4, E1399 residue of S1 and D1420 residue of S3 were disrupted in R1451C and R1451L channels. These hydrogen bonds were in part displaced toward the R3 residue of the S4, where R1454 now interacted with both E1399 and D1420 in R1451C and only with E1399 in R1451L channels (Fig. 5C).

## Discussion

The present study investigated the biophysical properties of two distinct substitutions in Na_V_1.4 of the same amino acid residue (R1451C and R1451L), found in individuals with three different and unusual clinical phenotypes. One individual carrying the R1451L substitution had NDM in between SCM and cold-aggravated PMC while the second patient had some episodes of paralysis similar to hyperPP and some to hypoPP. The individual carrying the R1451C mutation had a minor PP phenotype with one single episode of hypokalemic quadriplegia in a circumstantial glucocorticoid injection^8^. Our goal was to compare the functional defects of R1451L and R1451C mutant Na_V_1.4 channels expressed independently in tsA201 to determine whether a genotype-phenotype correlation may emerge from their biophysical properties.

The S4 segment contains several positively charged amino acids, including 5 arginine residues named R1 to R5, which are critical for the voltage sensitivity of VGSC. R1451 is identified as the R2 residue of domain IV for Na_V_1.4. Missense mutations associated to cold-induced PMC have been found on R1 (R1448C/H/P/S)^14–16^, while mutations leading to CMS-like phenotypes have been found on R3 (R1454W)^4^ and R4 (R1457H)^5^ residues of S4DIV. R1451C/L mutations slowed the current decay of fast inactivation and shifted the steady-state inactivation curve to hyperpolarized voltage as observed for all substitutions affecting arginine residues in this S4 segment. Unlike the R1454W and R1457H mutations leading to permanent muscle weakness, the recovery from fast inactivation and the frequency-dependent (data not shown) regulation of Na^+^ current were not affected. The decreased rate of fast inactivation exerts a gain of function effect, suggested to increase the AP duration to promote repetitive firing leading to myotonia and eventually to paralysis^1^. Accordingly, the decrease of fast inactivation rate was more pronounced for R1451L associated with the clinical continuum PMC (individual 2)/PP (individual 3). These individuals did not complain from myotonia in their daily life, but myotonic bursts were detected by EMG at room temperature. Furthermore, the observed increase in the window current may contribute to enhance the excitability, especially during a range of resting membrane potential.

Many skeletal muscle sodium channelopathies displayed abnormal episodes of muscle excitability induced by cold, including NDM associated with R1451L (individual 2). Nevertheless, we found that fast inactivation kinetics of R1451C/L were not more sensitive to temperature than the WT channels, as previously reported for R1448P^15^. Studies on cold-aggravated PMC-related mutations substituting R1 in S4DIV have shown divergent effects of temperature on channels properties depending upon the amino acid substitution. Fast inactivation kinetics of R1448C were reported slightly more temperature-sensitive than R1448P^14^, while no significant temperature sensitivity was observed for R1448H. The data support the threshold mechanism first proposed by Hayward *et al*., whereby the cold sensitivity is caused by attainment of a threshold of disruption by cold temperatures rather than changes in temperature sensitivity of mutant channels^17^. Interestingly, the kinetics of fast inactivation of R1451C were slower than those of R1451L at 10 °C, even if no cold-induced myotonia was reported for the individual carrying this mutation at the time of last examination.

The role of slow inactivation in PP is unclear. It has been proposed that a disruption of slow inactivation is necessary to account for the extended duration of paralysis in hyperPP^18^. Slow inactivation changes were expected for R1451C/L channels given that S4DIV arginines are important for slow inactivation mechanism^19^. Accordingly, our data showed an enhancement of slow inactivated state for R1451C/L as revealed by the slowed time constant of recovery. Again, this was more pronounced for R1451L. Enhancements of slow inactivation have been observed for R1454W and R1457H CMS-like related mutations. Amongst missense mutations associated with hyperPP, enhancement of slow inactivation was also observed for I1495F and the double mutant M1490L-M1493I channels^12,20^. We then propose that the combination of loss-of-function effects resulting from: (*i)* the hyperpolarized shift in steady state inactivation and *(i)* the enhancement of slow inactivation for the mutant channel would outweigh the gain of function effect of slowed entry into fast inactivation. This could result in paralytic attacks by haploinsufficiency resulting from the transiently reduced availability of Na_V_1.4. For example, the R1451L mutation shifted the voltage dependence of inactivation leftward by −19 mV: at a resting potential of −85 mV, about 60% of the mutant channels would be inactivated. The milder impact of R1451C on these parameters may account for the requirement of specific triggers (corticoid in addition to carbohydrate-rich meal) for inducing skeletal muscle paralysis. The reduced current density probably amplifies these loss-of-function effects resulting from impaired gating behavior. Missense substitutions resulting for some in a complete lack of Na^+^ current in heterologous cell expression system have been demonstrated as causing recessively-inherited fetal hypokinesia and congenital myopathy-like phenotype in humans^21^. The reduced Na^+^ current density reported here most likely results from defective trafficking of Na_V_1.4 at the membrane. Since little is known on the molecular interaction and the cell trafficking of Na_V_1.4 at the sarcolemma, determining the possible impact of R1451 substitutions is speculative, but certainly points on regions critical for the proper folding of Na_V_1.4^22,23^.

How do R1451C/L mutations affect the channel structure and lead to the functional defects observed in this study? The homology modeling provides a better understanding of the structural changes induced by these mutant channels by revealing a reorganization of the hydrogen bonds in the VSD of DIV. Given the importance of these counter charges interaction in the regulation of fast inactivation^24^, their disruption may explain one of the main biophysical properties affected by R1451C/L substitutions: the fast inactivated state. J. R. Groome and V. Winston experimentally demonstrated that neutralizing either the counter charge E1399 and D1420 in Na_V_1.4 as we observed within the R1451C/L mutant channels, is sufficient to slow the kinetic of fast inactivation^24^. Our model predicts that the salt bridge involving E1399 with R2 is displaced to R3 in both R1451C/L mutant while the salt bridge involving D1420 with R2 moves to R3 in R1451C and is lost in R1451L channels. Thus the alteration in salt bridges formation predicted by our homology model may support the defects observed in fast inactivation kinetics in R1451C/L. We propose that the complete loss of one of these salt bridges in R1451L may explain the stronger effect observed for this substitution. This may also explain the rare occurrence of paralytic events resulting from R1451C and the need of a trigger such as glucocorticoids, which have been proposed to induce hypoPP in individuals carrying susceptibility alleles by indirectly altering the activity of the Na^+^-K^+^ pump^8^.

In addition to the salt bridges reorganization in R1451C/L channels, S4DIV lost one important charged amino acid. *Capes et al*. showed that the neutralization of S4 charges of DIV eliminates most of the gating charge movement associated with DIV in Na_V_1.4. This results in a shift toward hyperpolarized voltages of the steady-state inactivation, slower kinetics of inactivation and less voltage dependence of these kinetics^25^. We suggest here that in R1451C/L, the loss of one charge affects the S4 movement and contributes to the observed impairment of inactivation.

This study focused on functional analysis of Na_V_1.4 in heterologous expression system and has showed biophysical defects in mutant channels that can explain NDM and hyperPP related to R1451 substitutions. Clinical continuity between NDM and hyperPP is well known with the existence of mixed phenotypes resulting from *SCN4A* mutations such as the A1156T (S4-S5DIII), and M1360V (S1DIV) amino acid substitutions that favor inactivation of the channel^26–28^. The association of PMC or hyperPP phenotypes with a single *SCN4A* substitution in the same family pointed on the critical role of modifying factors in the pathophysiological expression of a defective Na_V_1.4 gating behavior^26^. Our clinical observation suggests the existence of an overlap between the mechanistically unrelated hyperPP and hypoPP clusters. We could not demonstrate the segregation between the disease and the mutations. However, the *SCN4A* alleles encoding the R1451C and R1451L substitutions have been reported only for 2 and 1 on 112,664 sequenced alleles in the ExAC database respectively, rendering their presence in individuals suffering from the rare hypoPP disease due to chance very unlikely. Moreover, such a clinical overlap has been reported in a single family with heat-induced myotonia and cold-induced hypoPP segregating with P1158S substitution (S4S5DIII)^29^. That further underlines the critical role of additional factors on the phenotypic expression of some *SCN4A* mutations, together with the inter-individual phenotypic variability linked to R1451L.

The familial form of hypoPP results from substitutions of arginine residues in VSD of Na_V_1.4 or Ca_V_1.1 favoring one gating pore current with proton or monovalent cations leak. This omega current results in paradoxical depolarization of the resting membrane potential in low K^+^ through changes in ionic homeostasis resulting from the cumulated activities of K^+^ and chloride channels, ATPase pumps and NKCC transporters^30,31^. The lack of changes in the hydrophobic septum in our molecular model as well as previous functional studies showing that 3 arginine substitutions (R1, R2, and R3) are required to create a gating pore current through DIV of Na_V_1.4^32^ renders the gating pore mechanism in R1451L channel to account for hypoPP unlikely. As stated above, haploinsufficiency resulting from the transient but severe loss-of-function effect of the R1451 substitutions induced by challenging conditions may favor episodic muscle weakness as did the *SCN4a* null allele in heterozygous mice, but does not account for concomitant hypokalemia^33^. *CACNA1S* encoding Ca_V_1.1 was sequenced to exclude any mutation. Hypokalemic episodes of paralysis may also result from dominant-negative mutations of *KCNJ2* encoding Kir2.1 in the multisystemic Andersen-Tawil syndrome^34^. *KCNJ2* was sequenced to exclude any mutation in this gene. Furthermore, mutations in *KCNJ1*8 are susceptibility factors for thyrotoxic PP, most frequently observed in Asian males^35^. The possible implication of this potassium channel as modifying factor for the phenotypic expression of R1451 substitutions deserves further investigation.

In conclusion, we have characterized the effects of two distinct substitutions of the Na_V_1.4 R1451 residue associated with a clinical continuum ranging from PMC to hypoPP. We found that both mutations shift the steady-state inactivation to hyperpolarized potentials, enhance the slow inactivated state and slow the overall kinetics of fast inactivation. The effect was less pronounced for R1451C, which may explain its dependency on glucocorticoids to induce PP. Cooling further enhances the abnormalities of fast inactivation kinetics in mutant channels. Finally, we have shown that a dissimilar disruption of hydrogen bonds occurs in the VSD of R1451C/L and can account for the defects in gating behavior of these mutant channels. This functional work confirmed that substitutions of arginine residues in S4DIV result in unusual phenotypes of muscular sodium channelopathy that are especially sensitive to additional factors for their full clinical expressivity.

## Materials and Methods

Standard protocol approvals, registrations, and patient consents

All methods were carried out in accordance with relevant guidelines and regulations. The study had ethical approval from a national ethics committee (DC-2012-1535 and AC-2012- 1536). All patients gave written informed consent for genetic testing.

### DNA sequencing

Mutations in *SCN4A* were searched for by direct Sanger sequencing of all coding exons and flanking intron regions on genomic DNA extracted from blood samples. *CACNA1S and KCNJ2* exons were sequenced in all individuals to exclude mutations in these genes as causing the hypoPP phenotype. NM_000334.4 was used as reference for cDNA numbering of the *SCN4A* mutations.

### Cell cultures

TsA201 (Millipore Sigma, USA) cells were derived from HEK 293 cells by stable transfection with SV40 large T antigen^36^. The cells were grown under standard tissue culture conditions (5% CO_2_, 37 °C) in high-glucose DMEM supplemented with 10% FBS, 2 mM L-glutamine, 100 U/ml of penicillin, and 10 mg/ml of streptomycin (Thermo Fisher scientific, Canada). The cells were cotransfected using the calcium phosphate method in 10-cm petri dishes with the pRcCMV vector (5 μg) containing WT or mutant hNa_V_1.4 cDNAs and with the pIRES/GFP bicistronic vector (2.5 μg) containing β1-subunit cDNA. After 24-h incubation in the transfection solution, the cells were washed twice with PBS, trypsinized, and plated in 35-mm dishes with culture medium. Electrophysiological recordings were taken 48 h after the calcium phosphate treatment.

### Whole-cell patch-clamp recordings

The transfected cells were observed using a fluorescence microscope, and macroscopic Na^+^ currents were measured using the voltage-clamp mode of the whole-cell patch-clamp technique. Patch-clamp recordings were obtained using low-resistance, fire-polished electrodes (<1 MΩ) made from 8161 Corning borosilicate glass coated with Sylgard (Dow-Corning, USA) to minimize electrode capacitance. Currents were recorded using an Axopatch 200 amplifier and pClamp software (Molecular Devices, USA). The series resistance was 70–80% compensated. Whole-cell currents were filtered at 5 kHz, digitized at 10 kHz, and stored on a computer equipped with an analog-to-digital converter (Digidata 1300; Molecular Devices, USA). The cells were allowed to stabilize for 5 min (experiments at 22 °C) or 10 min (experiments at 10 °C and 15 °C) after the whole-cell configuration was established before recording the currents. Different equilibration periods were used because the currents took longer to stabilize at 10 °C and 15 °C. The temperatures were controlled using a bipolar temperature controller (Model TC-202; Warner, Canada) and were measured as close as possible to the cells using a bath sensor. Except where indicated, the currents were recorded at 22 °C.

The pipettes were filled with an intracellular solution composed of 35 mM NaCl, 105 mM CsF, 10 mM EGTA, and 10 mM Cs-HEPES. The pH was adjusted to 7.4 with 1 N CsOH. The external solution was composed of 150 mM NaCl, 2 mM KCl, 1.5 mM CaCl_2_, 1 mM MgCl_2_, 10 mM glucose, and 10 mM HEPES. The pH was adjusted to 7.4 with 1 N NaOH.

### Data analysis

The raw current traces were analyzed manually offline using Clampfit 10.2 (Molecular Devices). The resulting data were plotted using SigmaPlot 11.0 (SSPSS; USA) and Microsoft Excel. Peak currents were measured during a current-voltage protocol. Na^+^ current densities (pA/pF) were obtained by dividing the peak current by cell capacitance. Average I-V curves were obtained by plotting normalized peak currents or current densities against voltage. For the activation curves, Na^+^ conductance (G_Na_) was calculated from the peak current (I_Na_) using the following equation:

$${{\rm{G}}}_{Na}={{\rm{I}}}_{Na}/({\rm{V}}-{{\rm{E}}}_{Na})$$

where V is the test potential and E_Na_ is the reversal potential. Normalized G_Na_ values were plotted against the test potentials. For the inactivation curves, the peak current was normalized to the maximal value and was plotted against the conditioning pulse potential. The steady-state activation and inactivation curves were fitted to the following Boltzmann equation (equation 1):

1 $$G/{G}_{max}(\text{or}\,{I}/{I}_{{\max }})=1/[1+\exp ({V}_{1/2}-\,V/{k}_{v}]$$

where G is the conductance, Ι is the current, V_1/2_ is the voltage at which the channels are half-maximally activated or inactivated, and k_v_ is the slope factor. The time constant of channel fast inactivation *τ* was assessed by fitting a single or double exponential function to the current decay. To determine the entry to slow inactivation and the recovery from fast or slow inactivation, the test pulse peak current *I*_*test*_ was normalized to the corresponding prepulse current (*I*_*cont*_). *I*_*test*_/*I*_*cont*_ was plotted against the pulse interval. Recovery from fast or slow inactivation curves were fitted to the single (equation 2) and double exponential functions (equation 3) of the following equations, respectively:

2 $$I/{I}_{max}={A}_{fast}(1-\exp (-t/{\tau }_{fast}))$$

or

3 $$I/{I}_{max}={A}_{fast}(1-\exp (-t/{\tau }_{fast}))+{A}_{slow}(1-\exp (-{\rm{t}}/{\tau }_{slow}))$$

Data from the entry to slow inactivation curves were fitted to a double exponential decay function (equation 4) to determine the time constants:

4 $$I/{I}_{max}=y0\,+{A}_{slow}\exp (-t/{\tau }_{fast})+{A}_{fast}\exp (-{\rm{t}}/{\tau }_{slow})$$

where *τ*_*fast*_ and *τ*_*slow*_ are the time constants, *t* is the time, and *A*_*fast*_ and *A*_*slow*_ are the amplitudes of the time constants. The results are expressed as means ± SEM. Statistical significance was calculated using Student’s unpaired t-test, and the level of statistical significance was set at *P* < 0.05.

### Molecular dynamics simulations

To model the VSD of DIV, a standard MODELLER^37^ routine was used on our previously published Na_V_1.4 model^32^ with the difference that the rat sequence was replaced by the human sequence. The resulting VSD model was then inserted in a fully hydrated POPC bilayer. The system was equilibrated under normal constant temperature and pressure conditions (298 °K, 1 atm) in a 116 mM NaCl solution. To ensure the correct reorganization of the lipids and solution, the positions of the phosphates in the lipids and the positions of all the atoms in the channel were constrained during the first 0.5 ns. The protein was constrained for 2 ns. The harmonic constraints on the protein were progressively removed over 4 ns. Lastly, a 40-ns unrestrained molecular dynamics (MD) simulation of the entire channel was conducted to relax the system. The resulting model was used to generate the models for the R1451C and R1451L channels. The two models were relaxed using the same three-step technique described above for the WT channel model. All the MD simulations were carried out using NAMD2.9^38^. Langevin dynamics were applied to keep the temperature at 300 °K. The equations of motion were integrated using a multiple time-step algorithm^39^. Short- and long-range forces were calculated every first and second time-step, respectively, using 2.0-fs time-steps. Chemical bonds between hydrogens and heavy atoms were constrained to their equilibrium values. Long-range electrostatic forces were taken into account using the particle mesh Ewald approach^40^. The water molecules were described using the TIP3P model^41^. The simulations used the CHARMM27-CMAP force field with torsional cross-terms for the protein^42^ and CHARMM36 for the phospholipids^43^. A united-atom representation was adopted for the acyl chains of the POPC lipid molecules^44^. The simulations were performed on Université Laval’s Colosse supercomputer.

The putative salt bridges were mapped using the VMD Salt Bridges Plugin, Version 1.1. Potential salt bridges were mapped if the distance between oxygen atoms of acidic residues and nitrogen atoms of basic residues were within a 5 Å cut-off distance in a least one frame of the last 2 ns of the relaxation step. The distances reported are the average distances between the center of mass of the oxygens in the acidic side chain and the center of mass of the nitrogens in the basic side chain during the last 2 ns of system relaxation.
